# Taxonomic and functional shifts in the microbiome of severely obese, prediabetic patients: Ketogenic diet versus energy‐matched standard diet

**DOI:** 10.1111/dom.70364

**Published:** 2025-12-15

**Authors:** June Stone, Afroditi Tripyla, Melanie C. Scalise, Maria L. Balmer, Lia Bally, Dominik M. Meinel

**Affiliations:** ^1^ Institut für Bioanalytik Hochschule für Lifesciences, Fachhochschule Nordwestschweiz Muttenz Switzerland; ^2^ Department of Diabetes, Endocrinology, Nutritional Medicine and Metabolism Inselspital Bern University Hospital and University of Bern Bern Switzerland; ^3^ Diabetes Center Berne Bern Switzerland; ^4^ Institute for Infectious Diseases University of Bern Bern Switzerland; ^5^ Graduate School for Cellular and Biomedical Sciences University of Bern Bern Switzerland

**Keywords:** clinical trial, dietary intervention, energy regulation, obesity therapy

## Abstract

**Aims:**

Obesity and type 2 diabetes mellitus (T2DM) are among the leading global health challenges of the 21st century. While caloric restriction remains the cornerstone of weight loss interventions, ketogenic diets (KD), characterised by low carbohydrate and high fat intake, have been shown to improve metabolic health partly by modulating the gut microbiome. This study investigated the effects of a short‐term KD on gut microbiome composition and function in severely obese, prediabetic patients, compared to an energy‐matched standard diet (SD).

**Methods:**

In a randomised trial, patients with BMI >35 kg/m^2^ and prediabetes underwent either a 2‐week KD or isocaloric SD, both inducing a 30% energy deficit. Faecal samples collected before and after the intervention, alongside samples from healthy controls, were analysed by whole‐genome metagenomic sequencing.

**Results:**

At baseline, prediabetic patients exhibited greater interindividual variability and lower alpha diversity than healthy controls. KD resulted in a significant reduction of alpha diversity, largely driven by a selective loss of Lachnospiraceae, with a concomitant increase in Bacteroidaceae. Functional profiling revealed that KD, but not SD, altered genes coding for enzymes involved in energy metabolism, amino acid synthesis, nucleic acid activity, RNA modification, and vitamin biosynthesis. Additionally, serum acetate levels increased significantly following KD.

**Conclusions:**

These findings underscore that KD, independent of caloric intake, acutely remodels the gut microbiome's taxonomic and functional landscape, highlighting the microbiome as a potential mediator of KD's metabolic effects.

## 1 INTRODUCTION

In the last century, type 2 diabetes mellitus (T2DM) has become one of the leading causes of death worldwide, increasing by 70% in the last 20 years. T2DM is a prototypical diet‐related metabolic disorder, with dietary intake playing a central role in its pathophysiology. Further environmental risk factors for T2DM include age, lifestyle, stress, and microbiome health.^1^ The gut microbiome has been shown to serve as a crucial intermediary through which diet influences metabolic health.^2^ Diet is a major determinant of gut microbiome composition and function. In turn, the microbes modulate key metabolic pathways, including energy harvest, short‐chain fatty acid (SCFA) production, and host inflammatory response. Recent microbiome research has clarified how diet and lifestyle modulate key metabolic pathways relevant to T2DM.^1^

Ketogenic diet (KD) has been shown to not only aid in weight loss,^3^ but also alter microbiome composition and function.^4^ A standard Western diet consists of 45%–60% carbohydrates, 20%–30% fats, and 10%–35% protein, whereas KD consists of 70%–80% fat, 20%–25% protein, and 5%–10% carbohydrates.^3^ The lack of carbohydrate‐derived glucose in the blood leaves the cells in need of an alternative energy source. As the body enters a ‘state of ketosis’, it breaks down dietary fat into fatty acids, which are then transformed into ketone bodies by the liver. These ketone bodies act as the new energy source for the cells.^5^ In obese patients, the main effect of KD is thought to be weight loss.^3^ The decrease in carbohydrate intake further benefits the regulation of glucose tolerance and insulin resistance.^6^ There is also research suggesting that KD reduces inflammation and oxidative stress.^7^ The exact mechanisms of these effects are still being studied, particularly the role of the microbiome in producing metabolites such as SCFAs or other metabolically active substrates.^8^

Microbiome diversity and composition have been associated with T2DM, both as a contributing factor to the metabolic effects of the disease and as a treatment option (e.g., faecal transplant).^1^ Microbiota diversity is generally characterised using two parameters: alpha‐diversity reflects the number of microbial species present within a single sample and is therefore a measure of within‐individual richness. Beta‐diversity captures how different microbial communities are between individuals and thus quantifies inter‐individual variation. These metrics allow assessment of both the richness and the compositional variability of the microbiome in response to dietary interventions. Metabolites produced by gut bacteria play significant roles in immune response, carbohydrate metabolism, and insulin regulation.^9^ Butyrate, for example, regulates gut permeability, strengthening the host's first line of immune defence.^10^ Diet is one of the main factors influencing microbiome composition. Fibre, indigestible for humans, is a source of energy for many microbes; as such, fibre‐rich foods contribute to microbial diversity in the gut.^11^ While the prominent effect of diet on the microbiome is clear, the specific interactions are still being studied.

KD has been shown to be a powerful tool in modulating the microbiome. Alongside changes in the ratios of the two most prominent families within the gut—Bacteroidaceae and Lachnospiraceae,^12^ the diet shows anti‐inflammatory and anti‐oxidative effects, supports immune regulation, increases intestinal mobility, and strengthens barrier function.^13^ However, other studies indicated possibly adverse effects on colonic health, further emphasising that more studies are necessary to evaluate the health impacts of a KD beyond metabolism.^14^ In the here‐presented study, we aimed to determine the modulation of the microbiome in severely obese prediabetic patients under KD. Beyond assessing taxonomic alterations of the microbiome under KD, we examined whether changes in functional gene profiles correspond to shifts in microbial composition and metabolic capacity, relative to a calorically matched control diet.

## 2 METHODS

### 2.1 Study design, population, and procedures

This work included data from a randomised controlled trial, which was conducted at the Bern University Hospital in Switzerland and included patients aged ≥18 years with body mass index (BMI) >35 kg/m^2^ and prediabetes (defined as glycated haemoglobin [HbA1c] between 5.7% and 6.4%). Exclusion criteria were excess alcohol consumption (defined as >3 units/day for males, >2 units/day for females), moderate to severe kidney disease, nephrolithiasis, pregnancy, and breastfeeding. The study was conducted in accordance with Good Clinical Practice and the Declaration of Helsinki after local Ethics Committee approval (2019‐00383). All participants provided written informed consent prior to study‐related procedures. The study was registered on ClinicalTrials.gov (NCT03880162).

Participants were assigned to either KD or an energy‐matched standard diet (SD) (14 days) using minimization randomisation with BMI, age, and sex as stratifying variables. Both diets were designed to create a 30% energy deficit, calculated based on the daily energy requirements estimated using the Mifflin‐St Jeor equation^15^ and the estimated physical activity level. The KD provided 10% of energy from carbohydrates, 70% from fat, and 20% from protein, while the isocaloric SD consisted of 50% carbohydrates, 30% fat, and 20% protein. To accommodate individual meal preferences, participants received personalised daily diet plans specifying recipes, ingredients, and macronutrient composition for each meal. The daily fibre intake target was 25 g. Participants were also provided with a daily multivitamin supplement (Actilife all‐in‐one, Germany) and were instructed to drink 30–40 mL/kg of water daily. Participants who were on metformin were advised to stop the medication at least 14 days before diet initiation. Proton pump inhibitors were not stopped throughout the study period. Participants were instructed to perform a daily capillary ketone measurement (*β*‐hydroxybutyrate, *β*‐OHB) in a fasted state during the diet (Freestyle Precision Neo; Abbott, Chicago, Illinois, United States) and photo‐document their meals using a food‐diary app (See How You Eat Coach app, Healthy Revolution Ltd., Finland).

At day 0 and 14, participants were asked to collect stool samples (OMNIgene‐Gut‐OM‐200 tubes, DNA Genotek Inc., Ottawa, Canada), which were stored at −20°C. Before DNA extraction, 20 μL of Spike‐in Control I (Zymo Research, CA, USA) was added to a subset of the samples. Stool samples were also collected from healthy individuals, and results were compared to those of the severely obese patients with prediabetes.

### 2.2 DNA extraction and sequencing

DNA was extracted from stool samples using the DNEasy Powersoil Kit (QIAGEN, the Netherlands). Metagenome sequencing was performed according to the Nextera XT DNA library preparation protocol (Illumina, CA, USA). The resulting library was quality controlled, and instead of bead normalisation, sample normalisation was performed according to Qubit 4 Fluorometer quantification (Thermo Fisher, MA, USA). Samples were sequenced using a NextSeq 500/550 High Output Kit v2.5 (300 Cycles) cartridge on an Illumina NextSeq550 sequencer (Illumina).

### 2.3 SCFA measurement

SCFA concentrations in patient serum were quantified by liquid chromatography–tandem mass spectrometry (LC–MS/MS), following a protocol adapted from Liebisch et al.^16^ Briefly, 50 μL of serum was mixed with 50 μL of 70% isopropanol and homogenised. After homogenisation, samples were centrifuged at 16 000 × *g* for 10 min at 4°C. For derivatization, 50 μL of sample supernatant or quality control was combined with 50 μL of an internal standard mix containing [^13^C,^2^H₃]‐acetic acid, and 20 μL each of 200 mM 3‐nitrophenylhydrazine (3‐NPH) and 120 mM *N*‐(3‐dimethylaminopropyl)‐*N′*‐ethylcarbodiimide (EDC). The mixture was incubated at 40°C for 30 min and then quenched with 200 μL of 0.1% formic acid. After centrifugation, >200 μL of the reaction mixture was transferred to LC–MS vials for analysis.

LC–MS/MS was performed using a reverse‐phase C18 column with water (0.1% formic acid) and acetonitrile (0.1% formic acid) as mobile phases. A 4 μL injection volume was used. SCFA concentrations were quantified using a calibration curve generated from matrix‐matched standards and normalised to internal standards.

### 2.4 Data analysis

Data analysis was conducted using the ‘Microbial Genomics Module (version 21.1) on the CLC genomics workbench (version 21.0.3). After QC and trimming, the Illumina reads were matched to the 22GB QMI‐PTDB of June 2021 (QIAGEN), creating taxonomic abundance tables which served as a basis for alpha‐ and beta diversity analysis. For the functional analysis, a workflow was created to build a functional profile from the metagenomic reads, consisting of creating a reference metagenome with ‘de novo assembled metagenome’, followed by ‘find prokaryotic genes’ and ‘annotate CDS’. The individual reads were mapped back onto this reference. The contigs in the assembled genomic fragments were annotated with the Enzyme Commission (EC) database^17^ and the reads were mapped to the predicted enzymes and quantified. Coding DNA sequences reveal the potential metabolic activity of the microbiome.

### 2.5 Statistical analysis

Comparisons between the two groups were performed using two‐tailed unpaired t tests or non‐parametric equivalents if data were not normally distributed. Differences within each group pre‐ and post‐diet were assessed using two‐tailed paired t tests or non‐parametric equivalents if data were not normally distributed.

## 3 RESULTS

### 3.1 Baseline characteristics

This work included five participants in the KD group (49.2 ± 13.1 years, 3 females, BMI: 47.4 ± 7.9 kg/m^2^) and eight participants in the energy‐matched SD group (52.3 ± 10.1 years, 6 females, BMI: 43.0 ± 7.2 kg/m^2^). Groups were comparable in terms of weight, waist circumference, and body composition at baseline. Although randomisation was stratified by age, sex, and BMI, fasting insulin and HOMA‐IR were numerically higher in the KD group at baseline (Table 1). Given the small sample size, such metabolic variability is expected. All microbiome analyses were conducted as paired, within‐individual comparisons, thereby minimising the impact of baseline differences on the interpretation of microbiome‐related outcomes. Eleven healthy controls were also included (39.4 ± 14.3 years, 10 females, BMI: 22.6 ± 2.3 kg/m^2^). The macronutrient composition of the prescribed diets is shown in Supplementary Table 1. The mean fibre content in the 14‐day prescribed meal plan was 24.7 ± 3.7 g in the KD group and 26.5 ± 6.0 g in the SD group. *β*‐OHB levels were 0.4 ± 0.2 mmol/L and 0.1 ± 0.0 mmol/L in the KD and SD groups respectively, *p* = 0.004.

**TABLE 1:** Baseline characteristics.

Parameter	Ketogenic diet group (*n* = 5)	Energy‐matched standard diet group (*n* = 8)
Age, years	49.2 ± 13.1	52.3 ± 10.1
Females, *n* (%)	3 (60%)	6 (75%)
BMI, kg/m^2^	47.4 ± 7.9	43.0 ± 7.2
Weight, kg	134.2 ± 23.2	121.1 ± 28.5
Waist circumference, cm	137.8 ± 15.4	133.4 ± 16.4
Fat mass, kg	63.2 ± 15.5	51.8 ± 1.8
Fat free mass, %	53.2 ± 6.1	51.8 ± 4.3
HbA1c, %	6.4 ± 0.2	5.8 ± 0.6
Fasting glucose, mmol/L	5.4 ± 0.6	5.7 ± 0.6
Fasting insulin, pmol/L	184.3 ± 81.0	101.6 ± 26.9
HOMA‐IR	7.4 ± 3.3	4.4 ± 1.4
ASAT, U/L	21.8 ± 5.9	23.6 ± 4.8
ALAT, U/L	27.6 ± 6.5	26.1 ± 4.2
Creatinine, μmol/L	77.0 ± 23.2	74.1 ± 19.9
Obesity‐associated co‐morbidities, *n* (%)	Arterial hypertension: 5 (100.0%) Dyslipidemia: 3 (60.0%) Sleep apnea: 3 (40.0%)	Arterial hypertension: 5 (62.8%) Dyslipidemia: 7 (75.0%) Sleep apnea: 5 (37.5%)
Medication at baseline, *n* (%)	Metformin: 1 (20.0%)^a^ Antihypertensive: 3 (60.0%) Lipid lowering: 1 (20.0%) Antibiotic: 0 (0.0%) Proton pump inhibitor: 1 (20.0%)	Metformin: 3 (37.5%)^a^ Antihypertensive: 5 (62.8%) Lipid lowering: 3 (37.5%) Antibiotic: 0 (0.0%) Proton pump inhibitor: 2 (25.0%)

*Note*: Data are presented as mean ± standard deviation or *n* (percentage), as indicated.

Abbreviations: ALAT, alanine transaminase; ASAT, aspartate transaminase; BMI, body mass index; HbA1c, glycated haemoglobin; HOMA, Homeostatic model assessment for insulin resistance.

^a^ Metformin was stopped prior to study‐related procedures.

### 3.2 Ketogenic diet leads to a decrease in the alpha diversity of the microbiome

**FIGURE 1:**
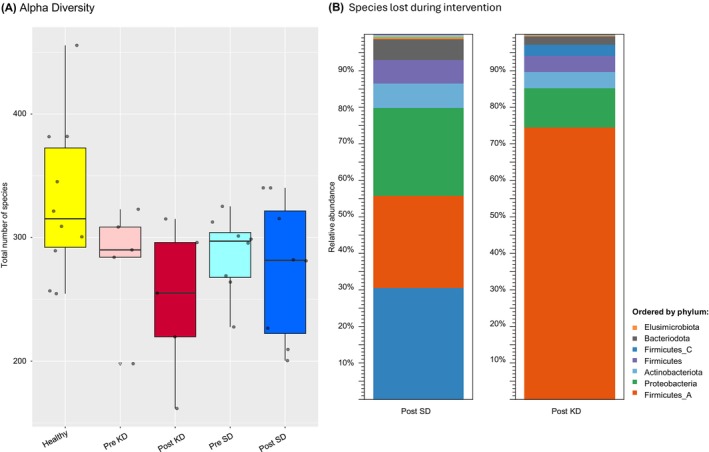
Alpha diversity analysis. (A) Box plot of absolute number of species within each intervention group, healthy (yellow), pre ketogenic diet (KD) (light red), post KD (dark red), pre standard diet (SD) (light blue) and post SD (dark blue). Healthy subjects show higher average alpha diversity (329.6), compared to prediabetic patient samples pre‐intervention (284.5) (Tukey HSD *p*‐value: 0.03). Alpha Diversity decreases from Pre KD (mean: 280.7) to post KD (mean: 249.5) (T. Test, paired, Tailed‐less, *p*‐value:0.14), whereas alpha diversity remains stable from pre SD (SD) (mean: 286.7) to Post SD (mean: 274.5). (B) Phylogeny of species lost during KD versus those lost during SD. Data contains combined species loss from each patient. Colour legend based on phylum, relative abundance includes all species lost from the belonging to corresponding phylum. Plot shows majority of species lost under KD belong to phylum Firmicutes_A. Further phyla include Proteobacteria, Firmicutes, Firmicutes_C, Actinobacteriota and Bacteroidota. Whereas under SD the distributions between Firmicutes_A, Firmicutes_C and Proteobacteria, and less prominent phyla Bacteroidota, Actinobacteriota and Firmicutes are more equal.

The alpha diversity analysis (Figure 1A) using absolute numbers of identified species per sample revealed significantly higher diversity in healthy subjects compared to the severely obese prediabetic patients prior to the intervention (*p* = 0.03). Additionally, alpha diversity decreased notably in the patients subjected to KD from 280.7 ± 43.6 to 249.5 ± 55, while the SD group remained largely unaffected from 286.8 ± 31.4 to 274.5 ± 56.6.

Noteworthy, the analysis showed that the species lost during SD and KD are different in distribution within the phylogenetic tree (Figure 1B). The species lost in the SD cohort are evenly distributed across several families of bacteria. Each of these families stems from different phylae, showing a large diversity on a phylogenetic level. However, almost 80% of the species lost during KD belong to the phylum Firmicutes_A, with almost 50% of the total species lost belonging to the family Lachnospiraceae (Figure 1B). This highlights that KD disproportionately impacts Lachnospiraceae, while the phylum Firmicutes_A maintains higher stability under SD, indicating a partially selective effect of KD on microbiome composition.

### 3.3 Ketogenic diet decreases lachnospiraceae and increases Bacteroidaceae

**FIGURE 2:**
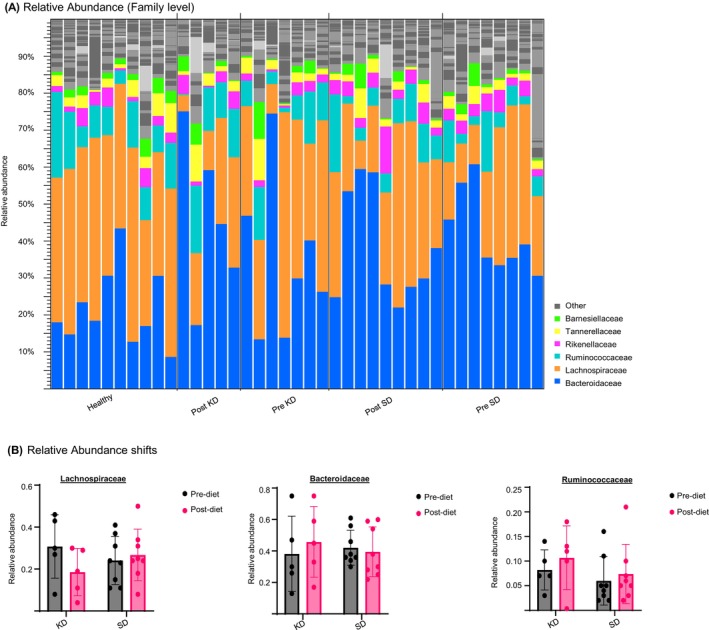
Relative abundance. (A) Relative family abundance of each individual prediabetic patient pre standard diet (SD), post SD, pre ketogenic diet (KD), post KD and healthy controls. The graph shows the most abundant families Bacteroidaceae, Lachnospiraceae and Ruminococcaceae. Prediabetic patients show high variability both pre and post intervention, whereas healthy controls show more homogenous profiles. (B) Comparison of relative abundances Pre KD and post KD versus pre SD and post SD of the three most abundant families Bacteroidaceae, Lachnospiraceae and Ruminococcaceae. Lachnospiraceae show a decrease from Pre KD to post KD, whereas the pre SD is unchanged compared to the post SD abundances. The Bacteroidaceae show a slight increase from pre KD to post KD and a minor decrease from pre SD to post SD. Ruminococcaceae show a small increase from pre‐KD to post KD and from pre SD to post SD. Lachnospiraceae are most affected by KD, with a visible decrease post intervention.

In the next step, the taxonomic distributions within each sample were analysed using relative abundance profiles (Figure 2A). A larger heterogeneity can be seen in the taxonomic abundances of the severely obese prediabetic patients compared to the healthy controls, indicating a more heterogeneous starting point for the microbiome of the prediabetic patients compared to the healthy controls. On the family level, KD leads to a decrease in Lachnospiraceae post‐intervention. KD further increased the relative abundances of Bacteroidaceae and Ruminococcaceae (Figure 2B). In contrast, SD intervention only minimally shifted the proportions of the three most abundant families, Lachnospiraceae, Bacteroidaceae, and Ruminococcaceae.

Detailed analysis on a species level (Supplementary Figure 2) showed that KD significantly decreased the abundances of two species belonging to the Lachnospiraceae family, such as *Roseburia* sp001940165 and *Eisenbergiella* sp900066775. Further Lachnospiraceae species, *Agathobacter rectalis*, also displayed a decreased abundance under KD. Additionally, species from the order Clostridia, CAG‐81 sp900066055, decreased in abundance, whereas its relative, CAG‐81 sp000435795, increased during KD. Finally, Bacteroides *F. pectinophilus*, a species belonging to the Bacteroidaceae, also significantly decreased in abundance post‐KD.

The SD induced no significant species abundance shifts. In summary, KD demonstrated a pronounced effect on the composition of the microbiome as compared to SD. In particular, the family Lachnospiraceae showed a clear decrease in abundance, and several of its species were significantly decreased post KD.

### 3.4 High variance beta diversity of severely obese prediabetic patients

**FIGURE 3:**
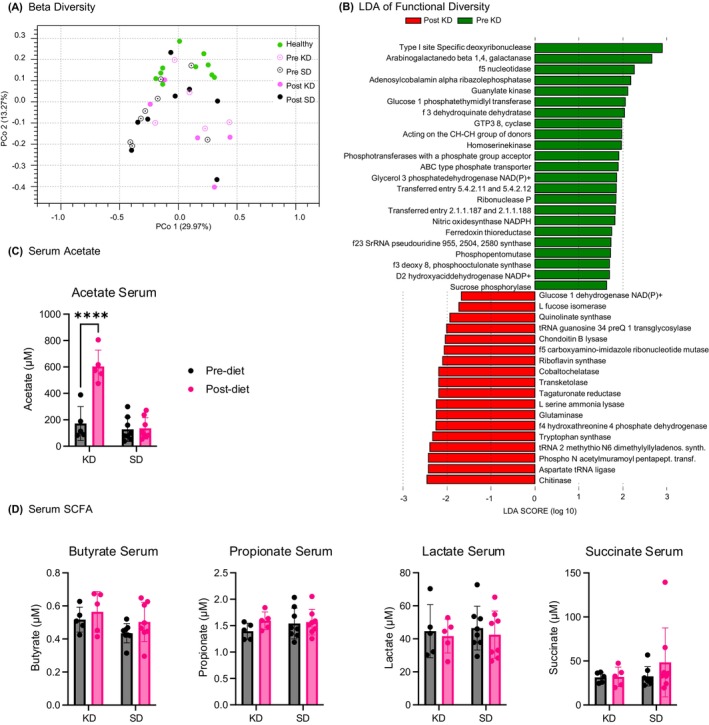
Effect of ketogenic diet on microbiome diversity and met bolic function. (A) Principal coordinate analysis (PCoA) scatterplot of beta diversity for each individual prediabetic patient pre standard diet (SD) (black circle), post SD (black dot), pre ketogenic diet (KD) (pink circle), post KD (pink dot) and healthy controls (green dot). Each point is labelled with a patient number, matching pre and post samples. PCo1 axis explains 29% of variance, PCo2 axis explains 13% of variance. The PCA shows no clear shift in any direction of either pre‐SD to post SD or pre KD to post KD. Prediabetic patient samples have been compared to the healthy controls, which cluster together, a high interpatient variance. (B) Genome derived functional potential linear discriminant analysis (LDA) results visualised in a vertical bar chart, shows enzyme commission (EC) terms which are significantly different in pre ketogenic diet (KD) versus post KD (Kruskal–Wallis (KW) sum‐rank test alpha value: 0.05, patient matched) with a logarithmic LDA score threshold for discriminative features of 1.5. Chart shows 23 EC terms downregulated under KD (green) and 18 EC terms upregulated under KD (red). (C) Acetate levels measured in serum pre and post diet intervention for the KD and SD groups. Levels significantly increased after the KD (multiple comparison test, adjusted *p*‐value: <0.0001 ****). (D) Butyrate, propionate, lactate and succinate levels measured in serum pre and post diet intervention for the KD and SD groups.

Beta diversity was analysed to allow for a systematic investigation of the changes in diversity within the groups. A principal coordinate analysis (PCoA) using the Bray–Curtis distance metric revealed high variance among the severely obese prediabetic patients (Figure 3A). This high variability can also be seen in the relative abundance table (Figure 2A). In comparison, the healthy patients showed less dispersity. This indicates that the microbial profiles of the healthy patients show higher similarity among each other than those of the severely obese prediabetic patients.

### 3.5 Functional analysis

The functional analysis revealed a detailed insight into the changes in metabolic activity of the microbiome induced by KD. The EC database^17^ was used to annotate the coding sequences in the microbial DNA with information on their predicted enzymatic activity. A linear discriminant analysis (LDA)^18^ was employed to identify EC terms significantly enriched under KD, indicating predicted shifts in enzymatic functions (Figure 3B). Notably, no significant alterations were observed in the control group maintained on SD. A detailed assessment of the EC terms enriched during KD (Table 2) categorised these enzymes according to their KEGG‐derived metabolic pathways, encompassing energy metabolism, amino acid metabolism, nucleic acid processes, RNA modification, and vitamin biosynthesis. The LDA findings demonstrate alterations in the genetic potential of energy metabolism that parallel the observed taxonomic shifts.

**TABLE 2:** Functional annotation of enriched and depleted genes under ketogenic diet.

Area of activity	KEGG description	EC Pathway	Enzyme	Terms	*p*‐Value	LDA score	Response to KD
Nucleic acid activity	Purine metabolism	ec00230	5‐(carboxyamino)imidazole ribonucleotide mutase	EC 5.4.99.18	0.028	2.061	↑
	Purine metabolism	ec00230	5′‐nucleotidase	EC 3.1.3.5	0.009	2.266	↓
	Purine metabolism	ec00230	Guanylate kinase	EC 2.7.4.8	0.016	2.122	↓
	Restriction enzyme		Type I site‐specific deoxyribonuclease	EC 3.1.21.3	0.047	2.899	↓
RNA modification	Methylation of tRNA		tRNA‐2‐methylthio‐N(6)‐dimethylallyladenosine synthase	EC 2.8.4.3	0.047	2.389	↑
	Aminoacyl‐tRNA biosynthesis	ec00970	Aspartate‐‐tRNA ligase	EC 6.1.1.12	0.047	2.423	↑
	Post transcriptional modification		tRNA‐guanosine(34) preQ(1) transglycosylase	EC 2.4.2.29	0.047	2.001	↑
	tRNA processing		ribonuclease P	EC 3.1.26.5	0.047	1.846	↓
	In vivo formation of 23S RNA pseudouridine746		23S rRNA pseudouridine746 synthase	EC 5.4.99.29	0.047	1.739	↓
	Methylates guanine748 at N1 in 23S rRNA		23S rRNA (guanine748‐N1)‐methyltransferase	EC 2.1.1.188	0.028	1.830	↓
Amino acid metabolsim	Arginine biosynthesis	ec00220	Glutaminase	EC 3.5.1.2	0.028	2.242	↑
	Glycine, serine and threonine metabolism	ec00260	L‐serine ammonia‐lyase	EC 4.3.1.17	0.009	2.238	↑
	Tryptophan synthesis		Tryptophan synthase	EC 4.2.1.122	0.028	2.325	↑
	Glycine, serine and threonine metabolism	ec00260	Homoserine kinase	EC 2.7.1.39	0.047	1.972	↓
	Arginine biosynthesis	ec00220	Nitric oxidesynthase (NADPH)	EC 1.14.13.39	0.028	1.827	↓
	Phenylalanine, tyrosine and tryptophan biosynthesis	ec00400	3‐dehydroquinate dehydratase	EC 4.2.1.10	0.028	2.047	↓
Energy metabolism	Reduction of 2‐oxocarboxylic acids		D‐2‐hydroxyacid dehydrogenase (NADP+)	EC 1.1.1.272	0.013	1.699	↑
	Fructose and mannose metabolism	ec00051	L‐fucose isomerase	EC 5.3.1.25	0.028	1.729	↑
	Pentose and glucuronate interconversions	ec00040	Tagaturonate reductase	EC 1.1.1.58	0.009	2.187	↑
	Pentose phosphate pathway	ec00030	Glucose 1‐dehydrogenase (NAD(P)+)	EC 1.1.1.47	0.028	1.667	↑
	Pentose phosphate pathway	ec00030	Transketolase	EC 2.2.1.1	0.016	2.185	↑
	Pentose phosphate pathway	ec00030	Phosphopentomutase	EC 5.4.2.7	0.047	1.729	↓
	Starch and sucrose metabolism	ec00500	Sucrose Phosporylase	EC 2.4.1.7	0.045	1.635	↓
	Glycolysis/glycine, serine and threonine metabolism	ec00010	Phosphoglycerate mutase (2,3‐diphosphoglycerate‐dependent)	EC 5.4.2.11	0.047	1.861	↓
Vitamin synthesis	Nicotinate and nicotinamide metabolism	ec00760	Quinolinate synthase	E.C. 2.5.1.72	0.047	1.932	↑
	Riboflavin metabolism	ec00740	Riboflavin synthase	EC 2.5.1.9	0.009	2.096	↑
	Vitamin B6 metabolism	ec00750	4‐hydroxythreonine‐4‐phosphate dehydrogenase	EC 1.1.1.262	0.016	2.246	↑
	Porphyrin metabolism	ec00860	Cobalt ochelatase	EC 6.6.1.2	0.016	2.184	↑
	Porphyrin metabolism	ec00860	Adenosylcobalamin/alpha‐ribazole phosphatase	EC 3.1.3.73	0.047	2.186	↓
	Folate biosynthesis	ec00790	GTP 3′, 8′ cyclase	EC 4.1.99.22	0.009	1.982	↓
Membrane activity	Peptidoglycan biosynthesis	ec00550	Phospho‐N‐acetylmuramoyl‐pentapeptide transferase	EC 2.7.8.13	0.016	2.417	↑
	Glycerophospholipid metabolism	ec00564	glycerol‐3‐phosphate dehydrogenase [NAD(P)+]	EC 1.1.1.94	0.016	1.864	↓
	Lipopolysaccharide biosynthesis	ec00540	3‐deoxy‐8‐phosphooctulonate synthase	EC 2.5.1.55	0.045	1.702	↓
	Phosphate‐specific transport system		ABC‐type phosphate transporter	EC 7.3.2.1	0.009	1.900	↓
Polymer lysis	Amino sugar and nucleotide sugar metabolism	ec00520	Chitinase	EC 3.2.1.14	0.047	2.453	↑
	Dermatan sulphate lysate		Chondroitin B lyase	EC 4.2.2.19	0.047	2.039	↑
	Hydrolyses beta bonds in type I plant arabinogalactans		Arabinogalactan endo‐*β*‐1,4‐galactanase	EC 3.2.1.89	0.028	2.665	↓
Electron transfer	Oxidoreductase		Ferredoxin thioredoxin reductase	EC 1.8.7.2	0.016	1.751	↓
Secondary metabolites	Biosynthesis of secondary metabolites	ec01110	Glucose‐1‐phosphate thymidylyl transferase	EC 2.7.7.24	0.047	2.058	↓

The production of SCFA, especially acetate, has been linked to metabolism in both rodents and humans, with some studies reporting a favourable impact on metabolic diseases.^19^ Measuring serum acetate levels before and after dietary intervention revealed a significant increase in acetate abundance after KD, while no change occurred upon SD (Figure 3C). Butyrate, propionate, lactate, and succinate levels were not different between groups (Figure 3D).

## 4 DISCUSSION

This study demonstrates that a short‐term KD profoundly alters the gut microbiome composition and its functional metabolic potential in severely obese, prediabetic patients, distinct from the effects of an energy‐matched SD. Specifically, KD reduced alpha diversity, selectively depleted members of the Lachnospiraceae family, increased Bacteroidaceae, and reshaped functional gene profiles linked to energy metabolism, amino acid synthesis, nucleic acid processes, and vitamin biosynthesis. These findings underline the capacity of macronutrient composition, independent of caloric content, to acutely remodel the microbiome in metabolic disease.

In line with prior work in children with epilepsy^5^, ^12^ and mouse models of neurodevelopmental disorders,^20^ KD here led to a significant decrease in alpha diversity, most likely due to reduced intake of fermentable fibres, the primary substrates sustaining diverse gut microbes.^11^, ^21^ Reduced diversity is a hallmark of gut dysbiosis linked to obesity and T2DM,^1^ and our baseline data confirmed that prediabetic patients already displayed lower alpha diversity than healthy controls. Of note, fasting insulin and HOMA‐IR were higher in the KD group at baseline. While this imbalance reflects the inherent variability in small clinical studies, it does not affect our microbiome analyses, which rely on paired comparisons within individuals. Nevertheless, we acknowledge that baseline metabolic differences may modulate clinical responses to dietary interventions and therefore interpret metabolic findings with caution.

Taxonomically, KD caused a pronounced decline in Lachnospiraceae—key butyrate producers dependent on complex carbohydrates^18^—and an increase in Bacteroidaceae, consistent with studies showing these taxa adaptively utilise host‐derived glycans under carbohydrate restriction.^22^ Although Lachnospiraceae reductions may mirror improved metabolic outcomes,^18^ their role remains complex, as they also maintain gut barrier integrity through SCFA production.^4^

Metagenomic functional profiling revealed that genes coding for enzymes in pathways tied to energy metabolism, amino acid turnover, RNA modification, and vitamin synthesis were significantly altered post‐KD. These results mirror observations by Lindefeldt et al.,^12^ who also found shifts in carbohydrate and nucleic acid metabolism after KD. Such functional changes may represent adaptive microbial strategies to altered substrate availability, potentially influencing host metabolic signalling.

Serum acetate increased significantly after KD, whereas butyrate, propionate, lactate, and succinate remained unchanged. This pattern is biologically plausible: acetate production is widely distributed across gut microbial taxa and does not rely primarily on Lachnospiraceae, whereas classical butyrate producers depend on fermentable carbohydrates that were markedly restricted under KD. Moreover, butyrate is absorbed by gut epithelial cells in significant amounts. Carbohydrate depletion may thus shift microbial fermentation pathways towards acetate generation. This selective SCFA response has been described in both rodent and human studies of carbohydrate restriction, where increased acetate can support lipid oxidation but has also been linked to lipogenesis and insulin resistance depending on metabolic context.^19^

Collectively, these findings emphasise that KD, beyond inducing weight loss and glycemic improvements,^23^ exerts distinct effects on gut ecology—effects that could be integral to its metabolic benefits or liabilities. However, the decline in microbial diversity warrants caution, as sustained loss of taxa may predispose one to long‐term dysbiosis.

This study is limited by its short duration and small sample size, precluding direct assessment of long‐term health impacts. Furthermore, while functional predictions based on metagenomic EC annotations provide insights into metabolic capacity, they do not confirm active metabolite fluxes. In summary, our findings show that macronutrient composition—independent of caloric intake—rapidly remodels both the taxonomic architecture and the functional gene repertoire of the gut microbiome in severely obese, prediabetic individuals. KD induced reproducible losses in fibre‐dependent taxa, expanded Bacteroidaceae, altered enzymatic pathways involved in energy and amino acid metabolism, and selectively increased circulating acetate. These acute microbiome shifts may contribute to the metabolic effects of KD, though the decline in microbial diversity warrants caution with longer‐term application. Larger and longer studies are required to define the durability of these changes and their mechanistic relevance for metabolic health.

## AUTHOR CONTRIBUTIONS

June Stone was responsible for analysing the sequencing data and writing the manuscript. Afroditi Tripyla was responsible for the clinical study and contributed her results to the report. Melanie C. Scalise was responsible for measuring and analysing the metabolic changes and contributing to the report. Maria L. Balmer, Lia Bally, and Dominik M. Meinel were responsible for supervising the work, acquiring funding, data interpretation, study design, and writing of the manuscript.

## CONFLICT OF INTEREST STATEMENT

To the best of our knowledge, there were no conflicts of interest in relation to this work.

## Supporting information


**Data S1:** Supple Fig 1: Distance matrix for Principal Coordinate Analysis (PCoA) of Beta Diversity.
**Supple Fig 2**: Relative abundance shifts under ketogenic diet.

## Data Availability

Raw read data is available via NCBI: PRJNA1333642.
