# Supplementary material for: Taxonomic and functional shifts in the microbiome of severely obese, prediabetic patients: Ketogenic diet versus energy‐matched standard diet
Source: Diabetes Obes Metab. 2025 Dec 15;28(3):1826–35. doi: 10.1111/dom.70364 (PMC12890762; doi:10.1111/dom.70364)
Supplement: Supplementary file 1 — Data S1: Supple Fig 1: Distance matrix for Principal Coordinate Analysis (PCoA) of Beta Diversity. Supple Fig 2: Relative abundance shifts under ketogenic diet. [file DOM-28-1826-s001.pdf]

Supplementary material:

1. Supplementary Table 1 Macronutrient composition of the prescribed diets
2. Distance matrix derived from the Principal Coordinate plot in Figure 3.
3. Collection of Species with LDA score >2 in pre vs post KD analysis, plotted as box and whisker plots, also showing SD pre and post values

Supplementary table 1: Macronutrient composition of the prescribed diets

Subject	Diet group	Energy (kcal)	Fat (g)	Saturated fat (g)	Protein (g)	Carbohydrates (g)	Fibers (g)
1	Low-carbohydrate	2496. 3 ± 75.8	195.2 ± 5.1	65.9 ± 10.7	117.9 ± 15.2	51.7 ± 9.1	28.8 ± 7.7
2	Low-carbohydrate	1693.4 ± 72.0	132.1 ± 7.7	37.0 ± 5.8	78.4 ± 7.7	35.2 ± 6.1	20.4 ± 5.8
3	Low-carbohydrate	1600.5 ± 63.7	119.4 ± 8.3	37.4 ± 10.0	83.1 ± 12.1	36.0 ± 7.2	21.2 ± 7.3
4	Low-carbohydrate	1773.3 ± 24.4	136.1 ± 5.4	43.2 ± 13.4	82.7 ± 7.2	38.7 ± 9.6	26.0 ± 10.7
5	Low-carbohydrate	1519.4 ± 24.8	112.8 ± 3.9	26.2 ± 6.3	75.8 ± 4.2	36.0 ± 2.3	27.3 ± 3.8
6	Standard	1912.3 ± 124.2	74.8 ± 10.3	20.6 ± 3.7	79.9 ± 8.0	202.0 ± 17.6	28.7 ± 4.8
7	Standard	1417.1 ± 97.9	52.9 ± 7.9	13.3 ± 2.8	65.8 ± 12.7	159.7 ± 21.7	19.9 ± 2.2
8	Standard	1104.2 ± 20.5	43.3 ± 5.0	13.1 ± 2.1	37.6 ± 11.0	132.9 ± 12.4	16.3 ± 3.3
9	Standard	1701.6 ± 106.1	61.7 ± 11.9	19.0 ± 3.3	69.8 ± 5.6	202.4 ± 11.8	28.5 ± 5.6
10	Standard	1701.6 ± 106.1	61.7 ± 11.9	19.0 ± 3.3	69.8 ± 5.6	202.4 ± 11.8	28.5 ± 5.6
11	Standard	1556.8 ± 27.2	53.6 ± 5.7	17.8 ± 4.0	73.5 ± 4.8	180.4 ± 5.3	27.0 ± 5.7
12	Standard	1873.7 ± 22.2	60.6 ± 3.1	18.6 ± 3.0	88.8 ± 4.8	228.8 ± 4.6	26.7 ± 5.1
13	Standard	2086.0 ± 41.8	66.3 ± 3.8	23.6 ± 5.0	98.9 ± 7.1	254.9 ± 4.7	36.3 ± 5.1

Data are presented as mean ± standard deviation (mean value during the 14-day prescribed meal plan).

		1	2	3	4	5	6	7	8	9	10	11	12	13	14	15	16	17	18	19	20	21	22	23	24	25	26	27	28	29	30	31	32	33	34	35	36
NXT-1	1	0.00	0.55	0.65	0.81	0.67	0.34	0.38	0.84	0.68	0.58	0.50	0.66	0.52	0.47	0.64	0.50	0.52	0.48	0.73	0.69	0.45	0.59	0.55	0.78	0.63	0.49	0.62	0.41	0.72	0.63	0.53	0.58	0.38	0.83	0.84	0.76
NXT-2	2	0.55	0.00	0.49	0.78	0.57	0.56	0.71	0.78	0.53	0.69	0.74	0.62	0.81	0.77	0.82	0.50	0.58	0.52	0.38	0.56	0.54	0.59	0.64	0.66	0.56	0.64	0.59	0.55	0.57	0.68	0.71	0.65	0.58	0.66	0.63	0.60
NXT-5	3	0.65	0.49	0.00	0.57	0.37	0.63	0.72	0.58	0.61	0.74	0.67	0.39	0.83	0.81	0.86	0.58	0.62	0.56	0.50	0.57	0.55	0.48	0.66	0.64	0.50	0.66	0.68	0.66	0.49	0.76	0.78	0.63	0.59	0.56	0.56	0.64
NXT-6	4	0.81	0.78	0.57	0.00	0.50	0.81	0.83	0.46	0.75	0.88	0.81	0.60	0.93	0.90	0.94	0.73	0.82	0.77	0.76	0.78	0.75	0.71	0.82	0.75	0.68	0.81	0.85	0.81	0.63	0.86	0.89	0.83	0.81	0.62	0.54	0.80
NXT-7	5	0.67	0.57	0.37	0.50	0.00	0.67	0.74	0.55	0.65	0.79	0.69	0.42	0.86	0.83	0.87	0.59	0.65	0.57	0.58	0.63	0.60	0.53	0.68	0.63	0.49	0.64	0.71	0.61	0.44	0.74	0.78	0.69	0.65	0.54	0.54	0.68
NXT-8	6	0.34	0.56	0.63	0.81	0.67	0.00	0.41	0.85	0.70	0.62	0.47	0.66	0.61	0.47	0.65	0.53	0.59	0.54	0.71	0.71	0.43	0.63	0.57	0.80	0.64	0.49	0.66	0.44	0.75	0.70	0.58	0.66	0.45	0.85	0.84	0.79
NXT-9	7	0.38	0.71	0.72	0.83	0.74	0.41	0.00	0.86	0.70	0.56	0.41	0.69	0.61	0.52	0.62	0.62	0.56	0.57	0.81	0.75	0.47	0.67	0.56	0.83	0.65	0.48	0.67	0.51	0.79	0.71	0.61	0.66	0.46	0.88	0.88	0.80
NXT-10	8	0.84	0.78	0.58	0.46	0.55	0.85	0.86	0.00	0.70	0.88	0.84	0.64	0.93	0.92	0.95	0.77	0.80	0.77	0.74	0.76	0.80	0.73	0.83	0.75	0.72	0.83	0.84	0.82	0.62	0.87	0.90	0.83	0.81	0.60	0.56	0.76
NXT-11	9	0.68	0.53	0.61	0.75	0.65	0.70	0.70	0.70	0.00	0.56	0.77	0.60	0.81	0.80	0.83	0.51	0.61	0.62	0.57	0.55	0.60	0.63	0.59	0.69	0.58	0.67	0.55	0.67	0.63	0.75	0.76	0.55	0.59	0.71	0.68	0.54
NXT-12	10	0.58	0.69	0.74	0.88	0.79	0.62	0.56	0.88	0.56	0.00	0.69	0.65	0.64	0.63	0.63	0.62	0.63	0.65	0.76	0.71	0.58	0.72	0.56	0.83	0.69	0.66	0.58	0.65	0.79	0.73	0.64	0.52	0.46	0.87	0.86	0.76
NXT-13	11	0.50	0.74	0.67	0.81	0.69	0.47	0.41	0.84	0.77	0.69	0.00	0.55	0.70	0.64	0.66	0.64	0.65	0.54	0.76	0.72	0.46	0.57	0.63	0.77	0.59	0.44	0.72	0.59	0.75	0.80	0.69	0.76	0.53	0.84	0.85	0.84
NXT-14	12	0.66	0.62	0.39	0.60	0.42	0.66	0.69	0.64	0.60	0.65	0.55	0.00	0.84	0.79	0.83	0.62	0.69	0.60	0.64	0.63	0.55	0.57	0.65	0.66	0.55	0.64	0.64	0.69	0.56	0.82	0.80	0.72	0.62	0.62	0.61	0.72
NXT-15	13	0.52	0.81	0.83	0.93	0.86	0.61	0.61	0.93	0.81	0.64	0.70	0.84	0.00	0.44	0.46	0.76	0.70	0.70	0.89	0.86	0.66	0.78	0.65	0.89	0.80	0.68	0.78	0.69	0.89	0.67	0.51	0.64	0.62	0.93	0.93	0.88
NXT-16	14	0.47	0.77	0.81	0.90	0.83	0.47	0.52	0.92	0.80	0.63	0.64	0.79	0.44	0.00	0.50	0.72	0.73	0.72	0.86	0.85	0.64	0.80	0.65	0.89	0.79	0.70	0.76	0.62	0.86	0.72	0.51	0.67	0.63	0.91	0.92	0.87
NXT-17	15	0.64	0.82	0.86	0.94	0.87	0.65	0.62	0.95	0.83	0.63	0.66	0.83	0.46	0.50	0.00	0.75	0.73	0.74	0.89	0.86	0.74	0.81	0.71	0.91	0.82	0.69	0.78	0.69	0.90	0.75	0.57	0.68	0.69	0.94	0.94	0.89
NXT-18	16	0.50	0.50	0.58	0.73	0.59	0.53	0.62	0.77	0.51	0.62	0.64	0.62	0.76	0.72	0.75	0.00	0.55	0.49	0.61	0.59	0.50	0.55	0.58	0.70	0.54	0.55	0.61	0.49	0.57	0.66	0.69	0.55	0.52	0.76	0.72	0.67
NXT-20	17	0.52	0.58	0.62	0.82	0.65	0.59	0.56	0.80	0.61	0.63	0.65	0.69	0.70	0.73	0.73	0.55	0.00	0.31	0.59	0.52	0.41	0.32	0.42	0.64	0.40	0.41	0.39	0.42	0.61	0.63	0.64	0.49	0.36	0.73	0.75	0.60
NXT-21	18	0.48	0.52	0.56	0.77	0.57	0.54	0.57	0.77	0.62	0.65	0.54	0.60	0.70	0.72	0.74	0.49	0.31	0.00	0.53	0.46	0.29	0.32	0.39	0.58	0.32	0.33	0.39	0.37	0.56	0.57	0.63	0.54	0.38	0.70	0.72	0.60
NXT-25	19	0.73	0.38	0.50	0.76	0.58	0.71	0.81	0.74	0.57	0.76	0.76	0.64	0.89	0.86	0.89	0.61	0.59	0.53	0.00	0.37	0.63	0.49	0.67	0.52	0.46	0.67	0.58	0.64	0.44	0.76	0.82	0.69	0.67	0.53	0.48	0.49
NXT-26	20	0.69	0.56	0.57	0.78	0.63	0.71	0.75	0.76	0.55	0.71	0.72	0.63	0.86	0.85	0.86	0.59	0.52	0.46	0.37	0.00	0.56	0.42	0.61	0.46	0.43	0.58	0.39	0.62	0.49	0.75	0.79	0.62	0.58	0.53	0.59	0.39
NXT-27	21	0.45	0.54	0.55	0.75	0.60	0.43	0.47	0.80	0.60	0.58	0.46	0.55	0.66	0.64	0.74	0.50	0.41	0.29	0.63	0.56	0.00	0.41	0.41	0.67	0.40	0.36	0.49	0.45	0.62	0.61	0.60	0.54	0.36	0.75	0.76	0.65
NXT-28	22	0.59	0.59	0.48	0.71	0.53	0.63	0.67	0.73	0.63	0.72	0.57	0.57	0.78	0.80	0.81	0.55	0.32	0.32	0.49	0.42	0.41	0.00	0.51	0.49	0.30	0.42	0.45	0.49	0.48	0.67	0.71	0.58	0.48	0.61	0.64	0.59
NXT-29	23	0.55	0.64	0.66	0.82	0.68	0.57	0.56	0.83	0.59	0.56	0.63	0.65	0.65	0.65	0.71	0.58	0.42	0.39	0.67	0.61	0.41	0.51	0.00	0.67	0.43	0.40	0.48	0.49	0.64	0.62	0.61	0.47	0.45	0.76	0.78	0.63
NXT-30	24	0.78	0.66	0.64	0.75	0.63	0.80	0.83	0.75	0.69	0.83	0.77	0.66	0.89	0.89	0.91	0.70	0.64	0.58	0.52	0.46	0.67	0.49	0.67	0.00	0.43	0.68	0.56	0.69	0.50	0.79	0.84	0.76	0.74	0.42	0.54	0.52
NXT-31	25	0.63	0.56	0.50	0.68	0.49	0.64	0.65	0.72	0.58	0.69	0.59	0.55	0.80	0.79	0.82	0.54	0.40	0.32	0.46	0.43	0.40	0.30	0.43	0.43	0.00	0.46	0.45	0.53	0.40	0.66	0.72	0.57	0.52	0.58	0.61	0.52
NXT-32	26	0.49	0.64	0.66	0.81	0.64	0.49	0.48	0.83	0.67	0.66	0.44	0.64	0.68	0.70	0.69	0.55	0.41	0.33	0.67	0.58	0.36	0.42	0.40	0.68	0.46	0.00	0.52	0.40	0.66	0.64	0.61	0.61	0.42	0.78	0.79	0.71
NXT-33	27	0.62	0.59	0.68	0.85	0.71	0.66	0.67	0.84	0.55	0.58	0.72	0.64	0.78	0.76	0.78	0.61	0.39	0.39	0.58	0.39	0.49	0.45	0.48	0.56	0.45	0.52	0.00	0.49	0.63	0.69	0.73	0.56	0.48	0.68	0.74	0.55
NXT-37	28	0.41	0.55	0.66	0.81	0.61	0.44	0.51	0.82	0.67	0.65	0.59	0.69	0.69	0.62	0.69	0.49	0.42	0.37	0.64	0.62	0.45	0.49	0.49	0.69	0.53	0.40	0.49	0.00	0.62	0.61	0.62	0.56	0.44	0.76	0.78	0.71
NXT-38	29	0.72	0.57	0.49	0.63	0.44	0.75	0.79	0.62	0.63	0.79	0.75	0.56	0.89	0.86	0.90	0.57	0.61	0.56	0.44	0.49	0.62	0.48	0.64	0.50	0.40	0.66	0.63	0.62	0.00	0.78	0.84	0.70	0.68	0.44	0.44	0.52
NXT-39	30	0.63	0.68	0.76	0.86	0.74	0.70	0.71	0.87	0.75	0.73	0.80	0.82	0.67	0.72	0.75	0.66	0.63	0.57	0.76	0.75	0.61	0.67	0.62	0.79	0.66	0.64	0.69	0.61	0.78	0.00	0.40	0.63	0.62	0.84	0.85	0.76
NXT-40	31	0.53	0.71	0.78	0.89	0.78	0.58	0.61	0.90	0.76	0.64	0.69	0.80	0.51	0.51	0.57	0.69	0.64	0.63	0.82	0.79	0.60	0.71	0.61	0.84	0.72	0.61	0.73	0.62	0.84	0.40	0.00	0.60	0.58	0.90	0.90	0.82
NXT-41	32	0.58	0.65	0.63	0.83	0.69	0.66	0.66	0.83	0.55	0.52	0.76	0.72	0.64	0.67	0.68	0.55	0.49	0.54	0.69	0.62	0.54	0.58	0.47	0.76	0.57	0.61	0.56	0.56	0.70	0.63	0.60	0.00	0.48	0.79	0.79	0.67
NXT-42	33	0.38	0.58	0.59	0.81	0.65	0.45	0.46	0.81	0.59	0.46	0.53	0.62	0.62	0.63	0.69	0.52	0.36	0.38	0.67	0.58	0.36	0.48	0.45	0.74	0.52	0.42	0.48	0.44	0.68	0.62	0.58	0.48	0.00	0.80	0.80	0.66
NXT-43	34	0.83	0.66	0.56	0.62	0.54	0.85	0.88	0.60	0.71	0.87	0.84	0.62	0.93	0.91	0.94	0.76	0.73	0.70	0.53	0.53	0.75	0.61	0.76	0.42	0.58	0.78	0.68	0.76	0.44	0.84	0.90	0.79	0.80	0.00	0.30	0.54
NXT-44	35	0.84	0.63	0.56	0.54	0.54	0.84	0.88	0.56	0.68	0.86	0.85	0.61	0.93	0.92	0.94	0.72	0.75	0.72	0.48	0.59	0.76	0.64	0.78	0.54	0.61	0.79	0.74	0.78	0.44	0.85	0.90	0.79	0.80	0.30	0.00	0.56
NXT-45	36	0.76	0.60	0.64	0.80	0.68	0.79	0.80	0.76	0.54	0.76	0.84	0.72	0.88	0.87	0.89	0.67	0.60	0.60	0.49	0.39	0.65	0.59	0.63	0.52	0.52	0.71	0.55	0.71	0.52	0.76	0.82	0.67	0.66	0.54	0.56	0.00

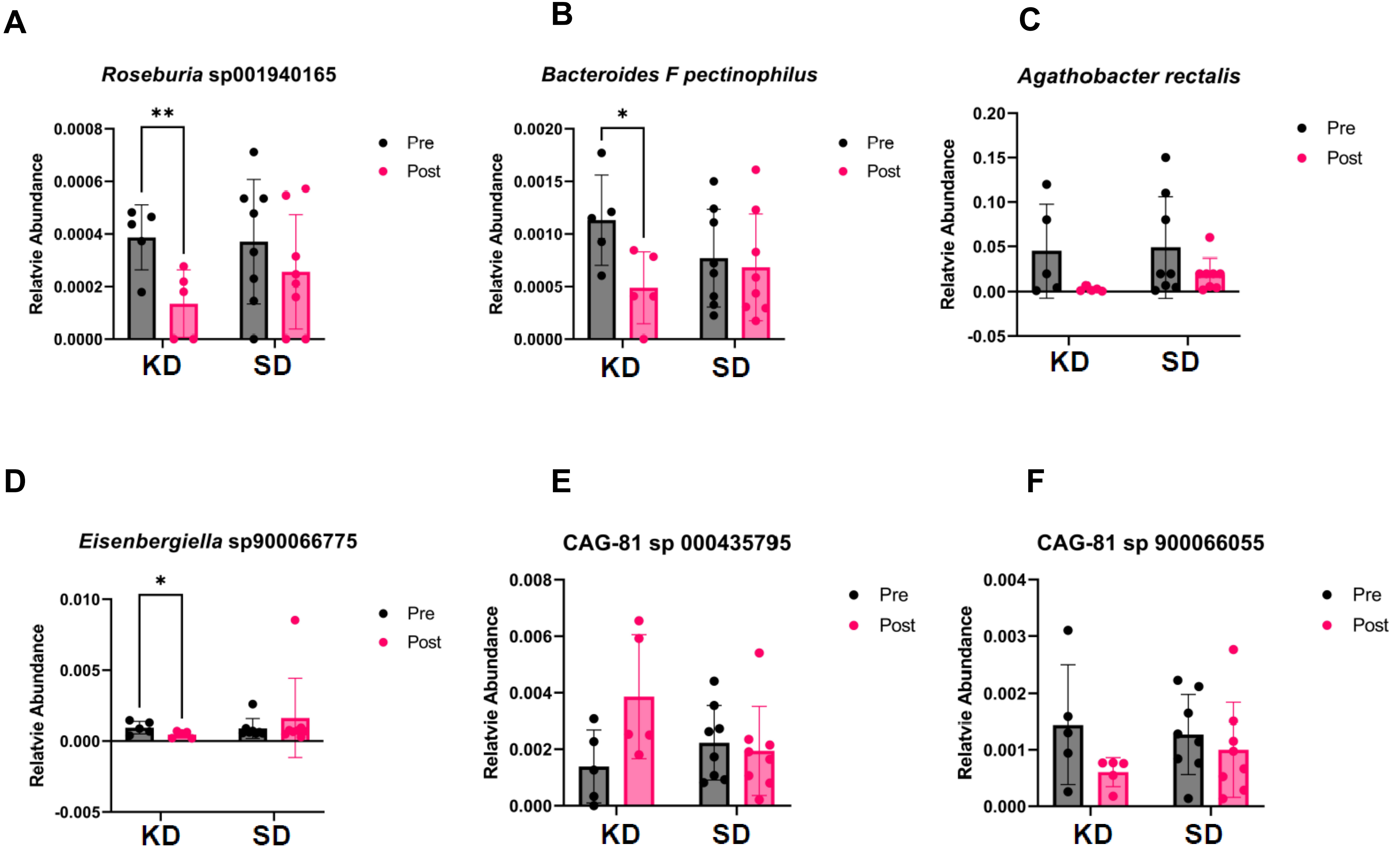
A
Roseburia sp001940165
**
0.0008
0.0006
0.0004
0.0002
0.0000
Relatvie Abundance
KD
SD
Pre
Post
B
Bacteroides F pectinophilus
*
0.0020
0.0015
0.0010
0.0005
0.0000
Relatvie Abundance
KD
SD
Pre
Post
C
Agathobacter rectalis
0.20
0.15
0.10
0.05
0.00
-0.05
Relatvie Abundance
KD
SD
Pre
Post
D
Eisenbergiella sp900066775
*
0.010
0.005
0.000
-0.005
Relatvie Abundance
KD
SD
Pre
Post
E
CAG-81 sp 000435795
0.008
0.006
0.004
0.002
0.000
Relatvie Abundance
KD
SD
Pre
Post
F
CAG-81 sp 900066055
0.004
0.003
0.002
0.001
0.000
Relatvie Abundance
KD
SD
Pre
Post
